# Durable and enhanced immunity against SARS-CoV-2 elicited by manganese nanoadjuvant formulated subunit vaccine

**DOI:** 10.1038/s41392-023-01718-8

**Published:** 2023-12-16

**Authors:** Mengyu Guo, Mingjing Cao, Jiufeng Sun, Ziwei Chen, Xin Wang, Lianpan Dai, George F. Gao, Yuliang Zhao, Yaling Wang, Chunying Chen

**Affiliations:** 1 CAS Key Laboratory for Biomedical Effects of Nanomaterials and Nanosafety & CAS Center for Excellence in Nanoscience, National Center for Nanoscience and Technology of China, Beijing, China; 2 Guangdong Provincial Institute of Public Health, Guangdong Provincial Center for Disease Control and Prevention, Guangzhou, China; 3 CAS Key Laboratory of Pathogen Microbiology and Immunology, Institute of Microbiology, Beijing, China

**Keywords:** Vaccines, Nanobiotechnology

**Dear editor**,

Vaccines are the most efficient and effective means to prevent infectious diseases, but improving the long-term protective efficacy is still a major challenge in contemporary vaccine development.^1^ The waning immunity varies depending on the diversification of the pathogen and the number of booster doses.^1^ Strategies to overcome this warrant is using adjuvants that amplify the immune response, and drive the production of memory B and T cells or long-lived plasma cells that recognize the pathogen for durable protection.^2–4^ Although existing adjuvants have achieved promising results, research on generating durable protective immunity is lacking in promoting vaccine development and staying ahead of global pandemics such as coronavirus disease 2019 (COVID-19). The precisely designed nanoadjuvants can enhance lymph node targeting and increase antigen-presenting cell (APCs) uptake, achieving the co-delivery of adjuvants and antigens and activating innate and adaptive immune responses.^5^ Previously, we reported a manganese nanoadjuvant (MnARK) and receptor-binding domain (RBD) monomer antigen formulated nanovaccine.^6^ MnARK transported antigens to lymph nodes, activated the STING pathway, elicited strong neutralizing abilities and increased immune memory T cell percentage against the infection of severe acute respiratory syndrome coronavirus 2 (SARS-CoV-2).^6^ Regarding the long-term protection potential of MnARK for subunit vaccine development, we further explored the durable immune regulation abilities of MnARK to a SARS-CoV-2 RBD dimer antigen, which has been used in an approved COVID-19 subunit vaccine ZF2001 with aluminum adjuvant (alum).^7,8^ TEM result revealed that RBD dimer could interact with BSA on MnARK surface and epitope can be well preserved (Supplementary Fig. 1a). The size and zeta potential of MnARK-RBD dimer nanovaccine was ~58 nm and -14 mV, respectively (Supplementary Fig. 1b, c).

To evaluate the immunogenicity of MnAKR-based nanovaccine, we intramuscularly (i.m.) immunized mice with 10 μg dose of RBD dimer formulated with MnARK or commercial alum adjuvant. The antibody (Ab) responses at various time points for up to 360 days after the prime vaccination were measured (Fig. 1a). The MnARK-based nanovaccine induced the most rapid Ab response, showing detectable anti-RBD antibodies (immunoglobulin M [IgM] and immunoglobulin G [IgG]) two weeks after the second vaccination (Fig. 1b, Supplementary Fig. 1d). The MnARK-based nanovaccine could elicit RBD-specific IgG up to endpoint titer of ~10^6^ after the third vaccination, which was significantly higher than that induced by the alum-based vaccine (*P* < *0.001*). Excitingly, durable and enhanced titers induced by MnARK were observed for the entire study (Fig. 1b). The Ab responses were similar from day 90 to day 360 across all animals receiving MnAKR-based nanovaccine. In contrast, a substantial response contraction was observed in animals immunized with alum-based vaccine. The IgG titer induced by the MnARK-based nanovaccine is 64 times and 46 times than that of the alum-based vaccine at day 240 and 360, respectively (Fig. 1b). Sera at day 56 and 360 were further tested for the neutralizing activities against live SARS-CoV-2 virus (prototype strain). Consistently, high levels of neutralizing antibody (NAb) were detected in RBD dimer-vaccinated mice using MnARK adjuvant, either at day 56 or 360 (Fig. 1c). MnARK-based nanovaccine outperformed alum-based vaccine to elicit high levels of NAbs with geometric mean titer (GMT) of 50% neutralization titer up to 2,353 at day 56. Encouragingly, at day 360, MnARK nanovaccine group maintained NAb titers at GMT of 512, 3 times than that of the alum-based vaccine group (GMT, 147) (Fig. 1c), suggesting the potential superiority of MnARK formulated nanovaccine in producing long-term immune responses.

**Fig. 1:**
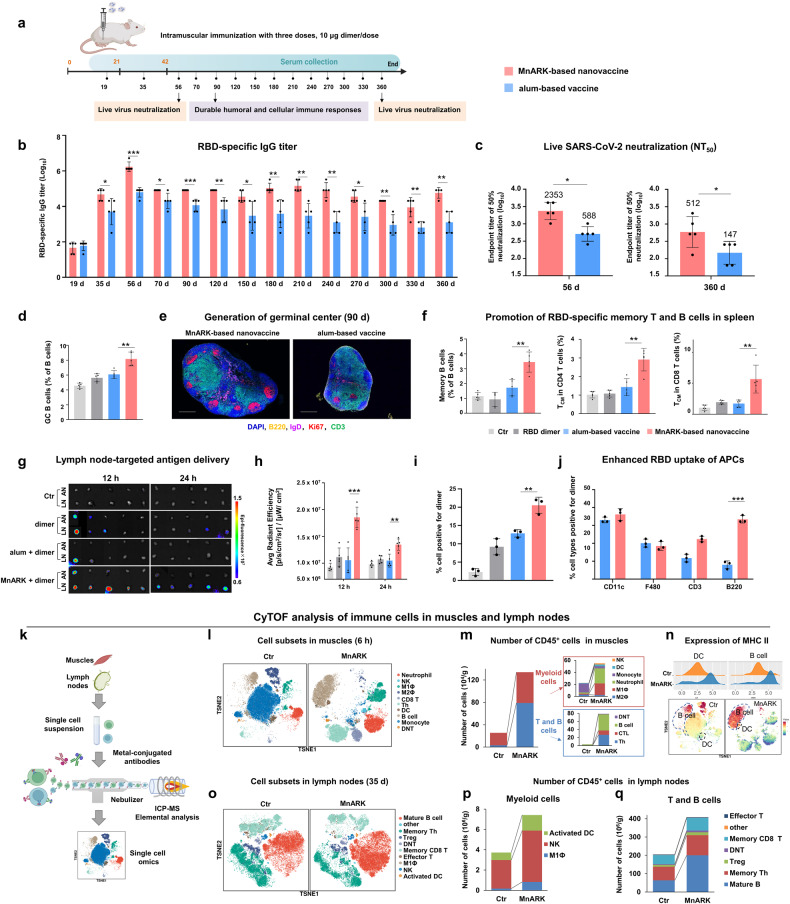
Enhanced durable humoral and cellular immune responses in MnARK-based subunit vaccine. **a** Schematic diagram of immunization and sample collection. BALB/c mice were immunized with 10 μg RBD dimer with MnARK (52 μg Mn) or alum (Alhydrogel adjuvant 2%, Croda, 52 μg Al) on days 0, 21, and 42. Sera were collected for up to 360 days. **b** anti-RBD IgG titers were measured by ELISA. Data are shown as the means ± SD (*n* = 5), and individual data are presented. **c** Sera collected at 56 days and 360 days post the first immunization were tested for neutralizing live SARS-CoV-2 (prototype strain). The NT_50_ was calculated as the reciprocal of serum dilution required for 50% neutralization of viral infection (n = 5). The values are the geometric mean titer (GMT). **d** The germinal center (GC) B cell percentages (n = 5) on day 56. **e** Representative immunochemistry staining of GCs in lymph nodes from different vaccinated groups on day 90 post-immunization. Anti-B220 (yellow), anti-IgD (purple), anti-Ki67 (red), anti-CD3 (green) and DAPI (blue) are presented. Bar = 200 μm. **f** The percentage of memory B cells and central memory (T_CM_) CD4^+^ and CD8 T^+^ cells in spleens (*n* = 5) on day 90. **g** Ex vivo images of lymph nodes at 12 h and 24 h post-injection (*n* = 5). Cy5-labeled RBD dimer or vaccines was intramuscularly injected into the right leg of mice. IN inguinal nodes; AN axillary nodes. **h** Quantification of the total fluorescence intensity (*n* = 5). **i** Quantify the percentage of dimer positive cells in lymph node samples (*n* = 3). **j** Cellular distribution of dimer uptake (measured by flow cytometry) among alum and MnARK group (*n* = 3). **k** CyTOF analysis of immune responses in muscles and lymph nodes of mice after i.m. injection with MnARK. Single cells obtained from muscles and lymph nodes were stained by antibody cocktails and analyzed by mass cytometry. **l** The t-SNE plot visualization of CD45^+^ cells pooled from 10,000 cellular events in muscles. **m** Number of CD45^+^ cell subsets in samples of muscles. The cell number of each population was calculated by multiplying the total number of isolated immune cells by the percentage of the population. **n** Normalized expression of the major histocompatibility complex class II (MHC II) marker on the t-SNE map. **o** The t-SNE plot visualization of CD45^+^ cells pooled from 10,000 cellular events in lymph nodes. **p**, **q** Number of CD45^+^ cell subsets (**p** myeloid cells, **q** T and B cells) in the lymph nodes sample. All the data were analyzed with a two-way ANOVA test and are presented as mean ± SD. *P < 0.05, **P < 0.01, ***P < 0.001

As establishing the germinal center (GC) response is critical for generating plasma cells and memory B cells capable of mediating durable protective immunity,^3,9^ we assessed whether the MnARK-based nanovaccine vaccination elicits a GC reaction for the capacity of responding B cells. We observed a striking increase in percentages of GC B cells two weeks after final vaccination with MnARK-based nanovaccine, demonstrating the advantages of MnARK over alum in promoting activation of antigen-specific B cells (Fig. 1d). Visualization of GCs in the lymph nodes remained detectable at least up to 90 days in animals vaccinated with MnARK relative to alum-based vaccine (Fig. 1e). This demonstrates that MnARK preferentially enhances the persistence of GC response, which is essential to the establishment of durable and broad immunity against virus and is a crucial metric of vaccine efficacy. Of note, MnARK-based nanovaccine induced a strikingly high level of memory B and T cells in spleens at day 90 post-vaccination (Fig. 1f). Memory B cells can be expanded and differentiated into antibody-secreting cells upon re-experience of antigen, playing a fundamental role in defending against viruses.^9,10^ Overall, these results indicate the durable SARS-CoV-2-specific immune response induced by MnARK-based vaccine, presenting deep profiling of anti-SARS-CoV-2 B cell responses.

To determine how MnARK influenced the immune response, we first vaccinated mice with Cy5-labeled RBD dimer *via* i.m. route to test the in vivo trafficking of antigen. MnARK induced an antigen depot comparable with that of alum at the injection sites (Supplementary Fig. 1e) and antigens undergo rapid metabolism through the kidneys and liver (Supplementary Fig. 1f). Exploiting the characteristics of albumin as a transporter and biotemplate, MnARK transported more RBD dimer to lymph nodes (Fig. 1g, h, Supplementary Fig. 2a), simultaneously promoting the uptake of APCs (Fig. 1i). Gating to firstly identify all dimer-positive cells, the result revealed that, among the RBD dimer-positive population in MnARK group, almost 36% were dendritic cells (DCs) and 33% were B cells, while an additional 20% were leukocytes of neither macrophages or T cells (Fig. 1j). DC and B cells seem to be nearly co-equal in their enhanced antigen uptake, associated with persistent activation of DCs (Supplementary Fig. 2b) and B cells (Fig. 1d–f). The stimulation of DCs by MnARK was evaluated using bone marrow dendritic cell (BMDC) in vitro. As shown in Supplementary Fig. 2b–e, MnARK-based nanovaccine significantly increased CD86^+^ and CD80^+^ expressions and pro-inflammatory cytokines levels compared with the free antigens and alum-based vaccine. And the higher the major histocompatibility complex class II/I (MHC II/MHC I)-presenting cells among the treated BMDCs demonstrated the enhanced cross-presentation of antigens. Of note, compared to alum, MnARK showed a 4-fold increase in the cellular uptake of RBD dimer in B cells (Fig. 1j), demonstrating the advantages of MnARK over alum in promoting activation of antigen-specific B cells. We used confocal microscopy to visualize antigen distribution in lymph nodes to confirm this flow cytometry analysis result. MnARK group can be seen in both DC and B cells (Supplementary Fig. 2a). Altogether, MnARK is superior to alum in increasing the cellular uptake of antigen in most APCs in lymph nodes, which play a crucial role in activating antigen-specific T and B cells.

To further investigate the features of MnARK-induced immunity, we performed mass cytometry (CyTOF) analysis of CD45^+^ cells in muscles and lymph nodes isolated from mice in the MnARK vaccinated group and the unvaccinated control group (Fig. 1k). In-depth studies of the 41 markers (Supplementary Table 1), expression profiles of immune cell clusters were visualized in a heatmap (Supplementary Fig. 2f), with 28 and 35 cell clusters identified in muscles and lymph nodes, respectively. The CD45^+^ subpopulations could be distinguished in the comprehensive single-cell visualization on t-distributed stochastic neighbor embedding (t-SNE) plots (Fig. 1l, o). Apparent alteration of immune cell subsets was observed in the MnARK vaccinated group compared with control group. Firstly, T cells, B cells and myeloid cells changed obviously in the muscles shortly at 6 h after injection (Fig. 1m). Specifically, DCs, B cells, T helper (Th) cells, cytotoxic T lymphocytes (CTL), neutrophils, and natural killer (NK) cells were recruited to the injection site. Notably, the expressions of the MHC II molecules on DCs and B cells were upregulated (Fig. 1n), which was beneficial for the antigen presentation. Moreover, in the lymph nodes at 35 d post immunization, we found significant expansion of activated DC cells, memory T cells and mature B cells (Fig. 1p, q). These results indicated that MnARK-based nanovaccine enhanced the magnitude of both humoral and CD8^+^ T cell responses. Consistent with the CyTOF results, the numbers of IFN-γ-secreting cells increased by 7.7-fold in the spleen than that of alum-based vaccine (Supplementary Fig. 2g). Sera from MnARK-based nanovaccine immunized animals showed significantly higher IgG2c-to-IgG1 titer ratio (Supplementary Fig. 2h) than that of alum-based vaccine. Correspondingly, intracellular cytokine staining (ICS) measurements of mouse splenocytes demonstrated that the MnARK-based nanovaccine induced more robust T_H_1 responses, which was shown by significantly higher frequencies of IFN-γ^+^, TNF-α^+^ and IL-2^+^ CD4^+^ T cell and CD8^+^ T cells, compared to alum-based vaccine (Supplementary Fig. 2i, j). Therefore, MnARK nanoadjuvant can induce a more robust T_H_1 cell response than alum adjuvant.

Our data showed steady maintenance of the antibody titer over 360 days through a judicious combination of antigen and MnARK adjuvant. Notably, MnARK generates potent CD8^+^ T cell responses, which are not optimally induced by commonly used alum adjuvants in vaccines. In addition, MnARK can target lymph nodes and enhance the uptake of antigen by B cells to promote B cell activation and prolong GC persistence, which presumably contribute to the magnitude and durability of humoral immunity (Supplementary Fig. 3). Several immunological outcomes in MnARK group could have contributed to successful induction of durable immune response: (1) promotion of antigen uptake by DCs and B cells (Fig. 1j), key cell types in sensing adjuvants to enhance antibody responses to vaccination. (2) successful induction of Th1 polarized CD4^+^ T cells (Supplementary Fig. 2g–j). The magnitude of the T helper cells migrate to the interface between the B cell follicle and the T cell area, where they stimulate antigen-activated B cells. And finally, 3) directly activating B cells of the adaptive immune system (Fig. 1n, q). Strategies to affect the molecular signatures of B cells by adjuvants is still limited. Further studies investigating the direct impact of MnARK on B cells could identify mechanisms that improve durable immune response.

### Supplementary information

SUPPLEMENTAL MATERIAL

## Data Availability

The data supporting the conclusion of this study can be required from the corresponding authors.
