# Correction: Durable and enhanced immunity against SARS-CoV-2 elicited by manganese nanoadjuvant formulated subunit vaccine

**DOI:** 10.1038/s41392-024-01739-x

**Published:** 2024-01-16

**Authors:** Mengyu Guo, Mingjing Cao, Jiufeng Sun, Ziwei Chen, Xin Wang, Lianpan Dai, George F. Gao, Yuliang Zhao, Yaling Wang, Chunying Chen

**Affiliations:** 1 CAS Key Laboratory for Biomedical Effects of Nanomaterials and Nanosafety & CAS Center for Excellence in Nanoscience, National Center for Nanoscience and Technology of China, Beijing, China; 2 Guangdong Provincial Institute of Public Health, Guangdong Provincial Center for Disease Control and Prevention, Guangzhou, China; 3 CAS Key Laboratory of Pathogen Microbiology and Immunology, Institute of Microbiology, Beijing, China

Correction to: *Signal Transduction and Targeted Therapy* 10.1038/s41392-023-01718-8, published online 16 December 2023

In this article^1^ Mengyu Guo, Mingjing Cao, and Jiufeng Sun are equally contributing authors. This information is presented in the accepted version of the manuscript, but was inadvertently overlooked during the production process.

The original article has been corrected.
