# Supplementary material for: Durable and enhanced immunity against SARS-CoV-2 elicited by manganese nanoadjuvant formulated subunit vaccine
Source: Signal Transduct Target Ther. 2023 Dec 16;8:462. doi: 10.1038/s41392-023-01718-8 (PMC10725496; doi:10.1038/s41392-023-01718-8)
Supplement: Supplementary file 1 — SUPPLEMENTAL MATERIAL [file 41392_2023_1718_MOESM1_ESM.pdf]

# Supplementary Materials for

## Durable and enhanced immunity against SARS-CoV-2 elicited by manganese nanoadjuvant formulated subunit vaccine

Mengyu Guo[1, #], Mingjing Cao[1, #], Jiufeng Sun[3, #], Ziwei Chen[1], Xin Wang[1], Lianpan Dai[2], George F. Gao[2], Yuliang Zhao[1], Yaling Wang[1, *], Chunying Chen[1, *]

[1]CAS Key Laboratory for Biomedical Effects of Nanomaterials and Nanosafety & CAS Center for Excellence in Nanoscience, National Center for Nanoscience and Technology of China, Beijing, 100190, China;

[2]CAS Key Laboratory of Pathogen Microbiology and Immunology, Institute of Microbiology, Beijing, 100101, China;

[3]Guangdong Provincial Institute of Public Health, Guangdong Provincial Center for Disease Control and Prevention, Guangzhou, 510440, China;

[#] These authors contributed equally to this work.

[*]Correspondence to: Yaling Wang (wangyl@nanoctr.cn); Chunying Chen (chenchy@nanoctr.cn)

**This PDF file includes:**

Materials and Methods

Figures. S1 to S3

Tables S1

## Materials and Methods

### Materials

RBD dimer was provided by George F. Gao's group.[1] MnARK adjuvant was generated as reported previously.[2] Alum adjuvant (Alhydrogel adjuvant 2%) was purchased from Croda. Anti-rabbit IgG-gold nanoparticles (5 nm) and anti-mouse IgG-gold nanoparticles (10 nm) were purchased from Sigma-Aldrich (Saint Louis, USA). Anti-RBD antibody was purchased from R&D Systems (Minneapolis, USA) and anti-BSA antibody was purchased from FineTest (China).

Bone marrow-derived dendritic cells (BMDC) were extracted from 10-week-old female BALB/c mice and were maintained in 1640 medium (Invitrogen, USA) at 37°C under 5% $CO_2$. The cell medium was supplemented with 10% fetal bovine serum (FBS), 1% penicillin-streptomycin (PS), 1% L-glutamine, 20 ng/ml GM-CSF (Peprotech) and 10 ng/ml IL-4 (Peprotech).

Specific pathogen-free (SPF) female BALB/c mice were purchased from Beijing Vital River Laboratory Animal Technology Co., Ltd. All mice used in this study are in good health and were housed under SPF conditions with a 12-hour light and dark cycle. All animal studies were carried out in accordance with the recommendations of the Guide for the Care and Use of Laboratory Animals. The mouse studies were conducted under the approval of the Institutional Animal Care and Use Committee of National Center for Nanoscience and Technology.

### Characterization of MnARK-RBD dimer nanovaccine.

The epitope of RBD on MnARK-RBD dimer nanovaccine was identified by

immunogold staining. The formulated vaccine sample was deposited onto a carbon-coated copper grid. The grid was blocked with 10% (w/v) goat serum in PBS and stained with 10 μL anti-rabbit BSA antibody (0.5 mg/mL) and 10 μL anti-mouse RBD antibody (0.5 mg/mL). After one hour of incubation, the sample was subsequently stained with 10 μL anti-rabbit IgG-gold nanoparticles (5 nm) and 10 μL anti-mouse IgG-gold nanoparticles (10 nm), both were diluted 1:20 with 10% (w/v) goat serum. After one hour of incubation, the sample was washed 5 times with PBS and then fixed in 10 μL 1% glutaraldehyde in PBS and washed 5 times with sterile water. Finally, the sample grid was examined using a TEM (HT7700, HITACHI, Japan). The position of 10 nm gold nanoparticles represents the RBD epitope.

**BMDC activation *in vitro***

BMDCs ($10^6$ cells per well) were plated onto 6-well plates. Free antigen, MnARK adjuvant, alum adjuvant, MnARK-based vaccine and alum-based vaccine were added to stimulate the cells and cultured at 37 °C for 24h. Cells were stained with anti-CD11c (Invitrogen, MR6F3), anti-CD80 (Biolegend, 16-10A1) and anti-CD86 (Biolegend, GL-1) antibodies at room temperature for 30 min. The BMDCs were washed with FACS buffer and analyzed using CytoFLEX flow cytometer (Beckman Coulter).

**Antigen internalization by APCs in lymph nodes**

Cy5 mono-reactive NHS ester (Thermo Fisher) was used to label the RBD dimer for imaging. BALB/c mice (6-8-week-old, n = 5) were intramuscularly injected with different vaccine formulations containing 10 μg Cy5-labeled dimer. Antigen persistence at the injection sites and lymph nodes was imaged and measured using an *in vivo*

imaging system IVIS (Perkin Elmer) (ex: 648 nm; em: 662 nm). Living image 4.5.2 Software was used to quantify fluorescence intensity at the injection sites and lymph nodes.

Lymph nodes were collected and ground to prepare single cells at 12 h after injection. The cells were strained through a 70 μm cell strainer and washed with PBS. Then, the cells were stained with anti-CD3 (Biolegend, 145-2C11), anti-CD11c (Biolegend, MR6F3), anti-F480 (Biolegend, BM8.1), anti-B220 (Biolegend, RA3-6B2) for 30 min at room temperature. After washing, cells were tested using CytoFLEX flow cytometer (Beckman Coulter).

Immunofluorescence staining of lymph node sections was prepared by GBA National Institute for Nanotechnology Innovation. Lymph node sections were treated following the same procedures as IHC, including dewaxing, rehydration, retrieval and 3% $H_2O_2$ treatment. After blocking with 5% BSA, slides were incubated with primary antibody anti-CD3 (Abcam, SP162), anti-CD11c (Abcam, D1V9Y), anti-F480 (Abcam, BM8) and anti-B220 (Abcam, Polyclonal) overnight. After staining with different secondary antibodies, slides were imaged via SlideViewer.

**Mouse immunization**

For immunization of mice, antigen was diluted with PBS, and mixed with MnARK or alum by a syringe. BALB/c mice were vaccinated with 10 μg RBD dimer per dose with MnARK (52 μg Mn per dose) or alum (52 μg Al per dose) through intramuscular injection, following an immunization schedule of one priming dose at week 0 plus two boosters at weeks 3 and 6. Serum samples were collected at days 19, 35, 56, 70, 90,

120, 150, 180, 210, 240, 270, 300, 330 and 360 via retro-orbital bleeding to measure the antibody titers.

For cellular immune response analyses, splenocytes were collected on day 56 after the first immunization for IFN-γ ELISPOT or ICS measurements. The lymph nodes were extracted from immunized mice on day 90 to characterize germinal center generation and analyzed by immunofluorescent. Splenocytes were collected on day 90 and analyzed by flow cytometry for memory immune response analyses.

**IgG antibody titer**

RBD-monomer protein was diluted to 3 μg/ml with ELISA coating solution (Solarbio), added to 96-well ELISA plate (Corning) and stayed overnight at 4 °C. Wells were blocked with 5% skim milk (BD) prepared with PBS at room temperature for 1 h. Serum samples were diluted with 5% skim milk from 20 times with 3 times gradient (1:20, 1:60, 1:180, 1:540, 1:1620, 1:4860, 1:14580, 1:43740, 1:131220). After washing with PBS, diluted serum samples were added and incubated at 37 °C for 2 hours. Then, samples were washed with PBST and incubated with goat anti-mouse IgG (Gene-Protein Link, P03S01M) with a 1/2,000 dilution at 37 °C for 1.5 hours. After washing with PBST 5 times, 60 μl TMB solution (Beyotime) was added. Finally, 2 M hydrochloric acid was added to terminate the reaction. A microplate reader was used to detect the reading value of OD450. The antibody titer is defined as the highest dilution times of serum, and the average reaction value should 2.5 times higher than that of the blank well.

A similar protocol with minor modifications was followed for IgM, IgG2c and

IgG1 ELISA analyses. Goat anti-mouse IgM-HRP (Gene-Protein Link, P03S01M), goat anti-mouse IgG2c-HRP (Abcam, ab97255) and goat anti-mouse IgG1-HRP (Abcam, ab97240) were used as secondary antibodies, respectively.

**Neutralization assay for live virus infection**

The live virus neutralization assay was conducted in a BSL-3 facility. Briefly, serum was collected on day 360 and serial 4-fold dilutions of serum were mixed with live SARS-CoV-2 (prototype strain), incubated at 37°C for 1 h, and added to the pre-plated Vero cells in 96-well plates. After that, inoculated plates were incubated at 37°C. The cytopathic effect (CPE) in each well was observed daily and recorded one week post-infection. The $NT_{50}$ was calculated as reciprocal of serum dilution required for 50% neutralization of viral infection.

**Cellular immunity in splenocytes**

Mouse splenocytes were obtained and plated at a $1 \times 10^6$ per well concentration. Cells were stimulated with a peptide pool of 20-mers (overlapping by 10 amino acids) spanning the SARS-CoV-2-S RBD for 12 hours at 37°C. Half of the cells were incubated with GolgiStop (BD Biosciences, USA) at the same time. Then, the cells were harvested and stained with anti-CD3 (Tonbo, 145-2C11), anti-CD4 (Tonbo, RM4-5), anti-CD8 (Tonbo, 53-6.7), anti-IL-2 (Biolegend, JES6-5H4), anti-IL-4 (Tonbo, 11B11), anti-TNF-α (Biolegend, MP6-XT22) and IFN-γ (Tonbo, XMG1.2). Cells without GolgiStop treatment were stained with B220 (Tonbo, RA3-6B2), GL7 (Biolegend, GL7), CD44 (Tonbo, IM7), CD62L (Tonbo, H1.2F3) and CD38 (Biolegend, 90). After a 0.5 h incubation, cells were collected, washed and tested with a CytoFLEX

flow cytometer (Beckman Coulter) and analyzed using FlowJo v10.8.1.

The antigen-specific T lymphocyte responses were evaluated by ELISPOT assay using mouse IFN-gamma ELISpot PLUS kit (ALP) (MabTech, 3321-4APT-2). Splenocytes were plated at a $2 \times 10^5$ per well concentration and stimulated with the peptide pool for 24 hours at 37°C. After incubation, the plates were processed with biotinylated IFN-γ detection antibody, streptavidin-HRP conjugate, and AEC substrate. When the colored spots were intense enough to be visually observed, the development was stopped by thoroughly rinsing samples with deionized water. The numbers of the spots were determined using an automatic ELISPOT reader.

**Mass cytometry and data analysis**

BALB/c mice were vaccinated with MnARK (52 μg per dose) or PBS (as the control group) through intramuscular injection, following an immunization schedule of one priming dose at week 0 plus a booster at week 3. Mass cytometry was conducted as previously described.[3] Single-cell suspensions were prepared from muscles and lymph nodes collected at day 35. Tissue samples from four mice from each group were pooled to obtain sufficient cells for reliable mass cytometry. Isolated cells were washed with PBS buffer and stained with cisplatin (Fluidigm) on ice to exclude dead cells, then cells were stained with surface antibody cocktail and intracellular antibody cocktail in order. The metal tag, provider and clone number of each antibody used in the mass cytometry are listed in Table S1. Antibody labeling with the metal tag was performed using the MaxPAR antibody labeling kit (Fluidigm). Conjugated antibodies were titrated for optimal concentration before use. After staining, cells run on the Helios 2 CyTOF

system (Fluidigm).

Mass cytometry raw data were normalized with the MATLAB versions of the normalization software.[4] FlowJo software was used to exclude dead cells, debris and adhesion cells, gating only live single immune cells ($CD45^+$ cells). The X-shift clustering algorithm was applied to identify cell subsets based on marker expression levels.[5] Cell types of each cluster were annotated according to the marker expression pattern on a heatmap of clusters and markers. The number of each cell population was calculated by multiplying the percentage of each defined population in the total $CD45^+$ cells by the number of isolated immune cells from tissues. The intensities of the markers were calculated using transformed median intensity values in each defined cell population. To visualize the high-dimensional data into two dimensions, the t-distributed stochastic neighbor embedding (t-SNE) dimensionality reduction algorithm was performed to show the distribution of each cluster and marker expressions.[6]

**Statistical analysis**

Values are presented as the mean with standard deviation (SD). Statistical significance among the different vaccination groups was calculated using the Student's t-test. $*p < 0.05$, $**p < 0.01$, $***p < 0.001$.

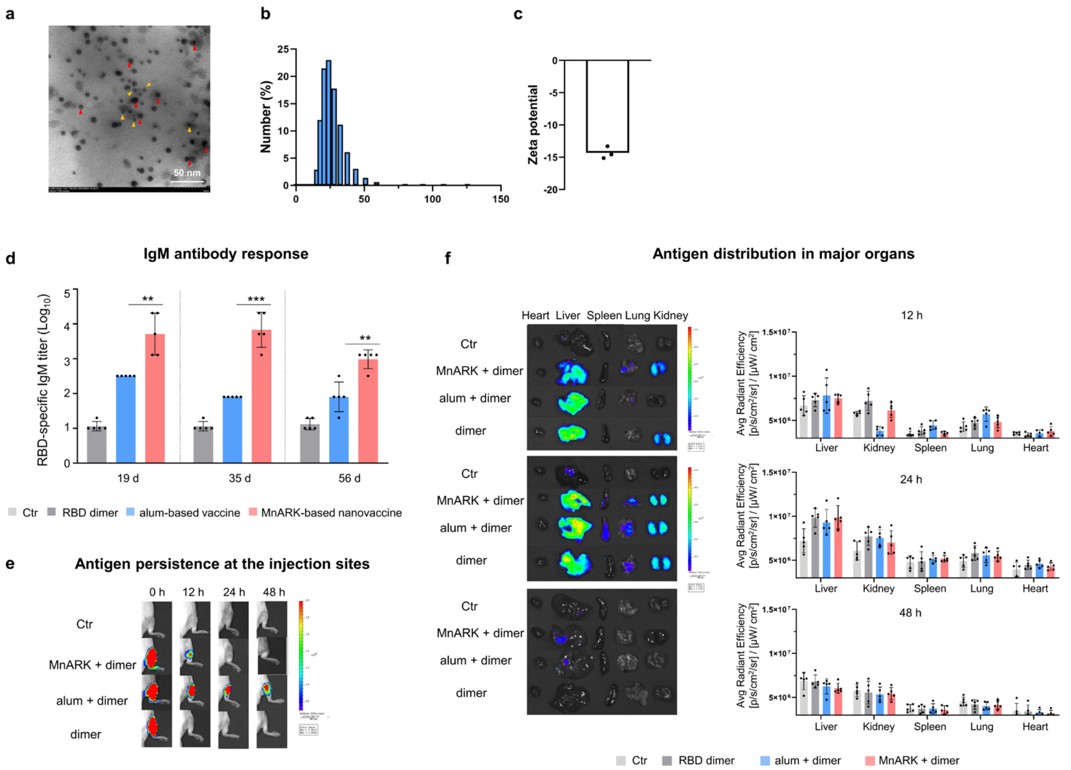


**Figure. S1. Characterization of MnARK-RBD dimer nanovaccine. a** TEM images of MnARK-RBD dimer formulation. The formulation was incubated with anti-mouse RBD antibody and anti-rabbit BSA antibody and then labeled with immunogold nanoparticles (red arrowheads, 10 nm anti-mouse IgG-gold nanoparticles bound anti-RBD antibody; yellow arrowheads, 5 nm anti-rabbit-IgG-gold nanoparticles bound anti-BSA antibody). **b** The hydrodiameter of MnARK-RBD dimer nanovaccine. **c** The zeta potential of MnARK-RBD dimer nanovaccine. **d** IgM antibody response induced by vaccine candidates in mice at day 19, 35 and 56. BALB/c mice were immunized with 10 μg RBD dimer with 52 μg MnARK or alum (Alhydrogel adjuvant 2%, Croda) on day 0, 21 and 42. Serum samples were collected after each immunization (19 days after 1st immunization, 14 days after 2nd immunization, and 14 days after 3rd immunization) to evaluate the IgM antibody response (n = 5). **e** Antigen persistence at

the injection sites. Cy5-labeled RBD dimer or nanovaccine was intramuscularly injected into the right leg of mice. The injection sites were imaged using an *in vivo* imaging system IVIS. **f** Representative IVIS images of major organs at 12 h, 24 h and 48 h post-injection and quantification of the fluorescence intensity (n = 5).

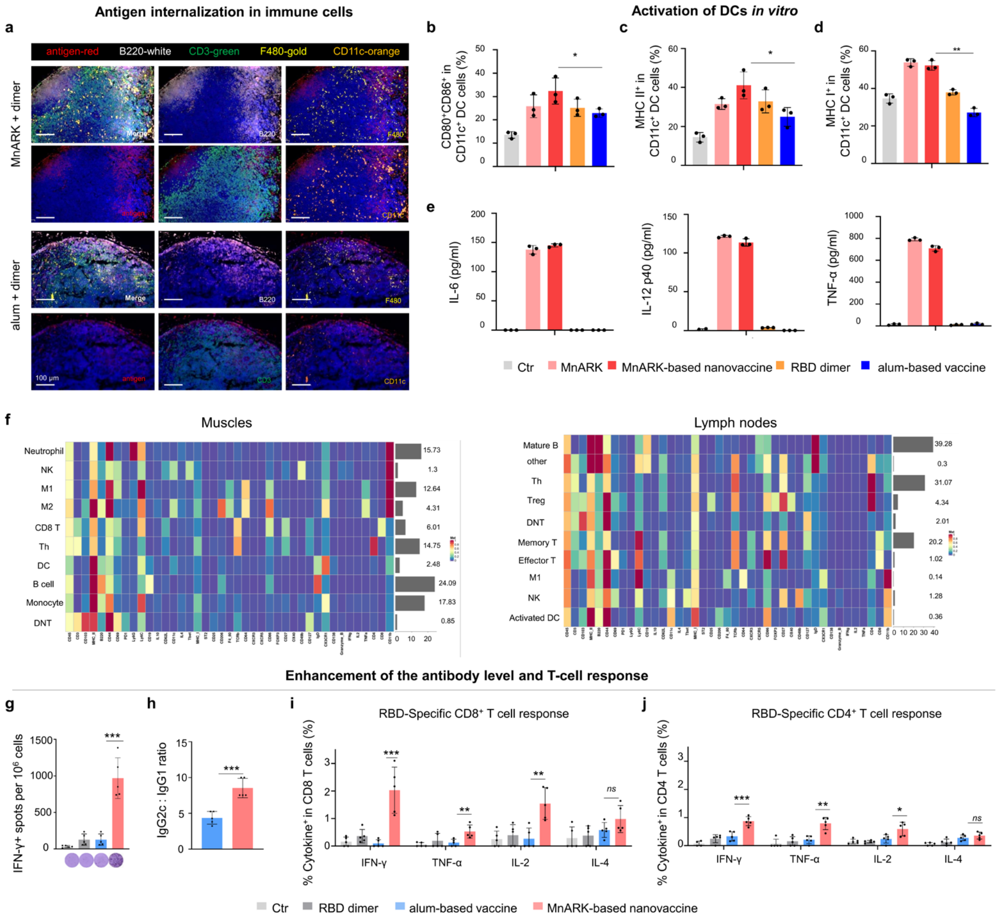


**Figure. S2. Enhancement of the antigen internalization and T-cell response by the MnARK nanovaccine. a** Immunofluorescence image of dimer internalization by different immune cells in lymph nodes**.** Antigen (red), B cell (white), T cell (green), macrophage (gold), DC (orange). **b-d** Activation of BMDCs by the MnARK nanovaccine. The expression of CD86, CD80, MHC II and MHC I on the surface of BMDCs were analyzed by flow cytometry. **e** Inflammatory cytokine level (IL-6, IL-

12p40, TNF-α) was measured by ELISA. **f** Heatmaps showing normalized expression of 41 surface markers in samples of muscles and lymph nodes**.** Relative frequencies are shown as a color bar at the right panel. **g-j** Enhancement of the antibody level and T-cell response by the MnARK nanovaccine. BALB/c mice were immunized with 10 μg RBD dimer with 52 μg MnARK or alum (Alhydrogel adjuvant 2%, Croda) on day 0, day 21 and day 42. Serum samples and splenocytes were collected 14 days after 3rd immunization (n = 5). **g** IFN-γ production measured by ELISPOT. **h** IgG2c : IgG1 ratio. **i, j** Flow cytometry analysis of $CD8^+$ and $CD4^+$ T cells, which express IFN-γ, TNF-α, IL-2 and IL-4. Statistical significance was tested with a two-tailed, unpaired Student's t-test.

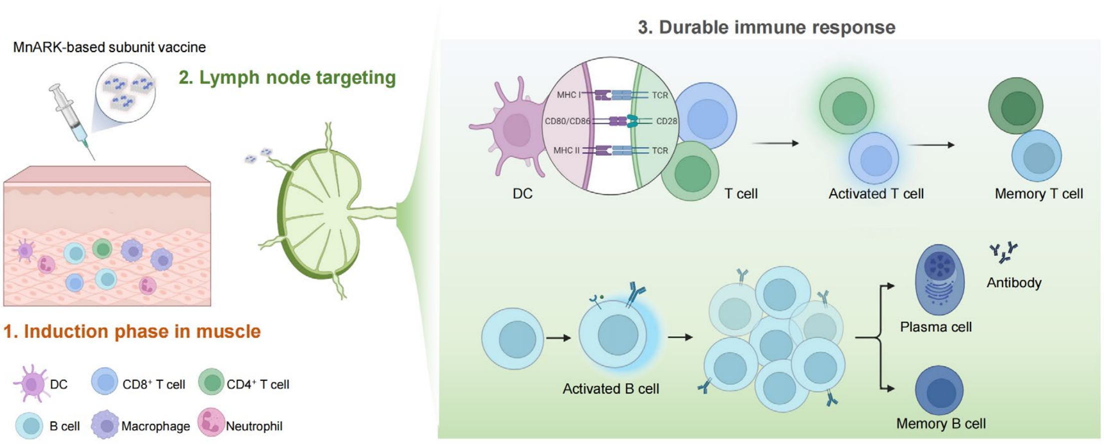


**Figure. S3. The MnARK-based nanovaccine efficiently targets lymph nodes, stimulates DC activation to promote cellular immunity and activates B cells to generate a durable humoral response. Created by Biorender.**

**Table S1. Details of antibodies used in the CyTOF analysis**

| No. | Label | Antibody | Clone | Vender | Position |
|---|---|---|---|---|---|
| **1** | 89Y | CD45 | 30-F11 | Biolegend | surface |
| **2** | 115ln | CD3ε | 145-2C11 | Biolegend | surface |
| **3** | 141Pr | CD103 | 2E7 | Biolegend | surface |
| **4** | 142Nd | MHC II | M5/114.15.2 | Biolegend | surface |
| **5** | 143Nd | B220 | RA3-6B2 | Biolegend | surface |
| **6** | 144Nd | CD44 | IM7 | Biolegend | surface |
| **7** | 145Nd | CD69 | H1.2F3 | Biolegend | surface |
| **8** | 146Nd | CD279 | 29F.1A12 | Biolegend | surface |
| **9** | 147Sm | Ly-6G | 1A8 | Biolegend | surface |
| **10** | 148Nd | Ly-6C | HK1.4 | Biolegend | surface |
| **11** | 149Sm | CD19 | 6D5 | Biolegend | surface |
| **12** | 150Nd | IL-10 | JES5-16E3 | Biolegend | intra |
| **13** | 151Eu | CD62L | MEL-14 | Biolegend | surface |
| **14** | 152Sm | CD11c | N418 | Biolegend | surface |
| **15** | 153Eu | IL-4 | 11B11 | Biolegend | intra |
| **16** | 154Sm | T-bet | 4B10 | Biolegend | intra |
| **17** | 155Gd | MHC I | 28-14-8 | Biolegend | surface |
| **18** | 156Gd | IL-33Rα | DIH9 | Biolegend | surface |
| **19** | 157Gd | CD25 | 3C7 | Biolegend | surface |
| **20** | 158Gd | CD206 | C068C2 | Biolegend | intra |

| | | | | | |
|---|---|---|---|---|---|
| **21** | 159Tb | F4/80 | Cl:A3-1 | BioRAD | surface |
| **22** | 160Gd | TCR β chain | H57-597 | Biolegend | surface |
| **23** | 161Dy | CD64 | X54-5/7.1 | Biolegend | surface |
| **24** | 162Dy | CD183 | CXCR3-173 | Biolegend | surface |
| **25** | 163Dy | CD185 | L138D7 | Biolegend | surface |
| **26** | 164Dy | CD86 | GL-1 | Biolegend | surface |
| **27** | 165Ho | FOXP3 | FJK-16s | eBioscience | intra |
| **28** | 166Er | CD27 | LG.3A10 | Biolegend | surface |
| **29** | 167Er | CD134 | OX-86 | Biolegend | surface |
| **30** | 168Er | CD49b | DX5 | Biolegend | surface |
| **31** | 169Tm | CD127 | A7R34 | Biolegend | surface |
| **32** | 170Er | IgD | 11-26c.2a | Biolegend | surface |
| **33** | 171Yb | CX3CR1 | SA011F11 | Biolegend | surface |
| **34** | 172Yb | CD138 | 281-2 | Biolegend | surface |
| **35** | 173Yb | Granzyme B | GB11 | Fluidigm | intra |
| **36** | 174Yb | IFN-γ | XMG1.2 | Bio-Xcell | intra |
| **37** | 175Lu | IL-2 | JES6-5H4 | Biolegend | intra |
| **38** | 176Yb | TNF-α | MP6-XT22 | Biolegend | intra |
| **39** | 197Au | CD4 | RM4-5 | Biolegend | surface |
| **40** | 198pt | CD8a | 53-6.7 | Biolegend | surface |
| **41** | 209Bi | CD11b | M1/70 | BioLegend | surface |

1

# References


1 Dai, L. *et al.* A universal design of Betacoronavirus vaccines against COVID-19, MERS, and SARS. *Cell* **182**, 722-733 (2020).

2 Wang, Y. *et al*. Engineering a self-navigated MnARK nanovaccine for inducing potent protective immunity against novel coronavirus. *Nano Today* **38**, 101139 (2021).

3 Han, G. *et al*. Metal-isotope-tagged monoclonal antibodies for high-dimensional mass cytometry. *Nat. Protoc.* **13**, 2121-2148 (2018).

4 Mei, H., Leipold, M. D. & Maecker, H. T. Platinum-conjugated antibodies for application in mass cytometry. *Cytometry Part A* **89A**, 292-300 (2016).

5 Samusik, N. *et al.* Automated mapping of phenotype space with single-cell data. *Nat. Methods* **13**, 493-496 (2016).

6 van der Maaten, L. & Hinton, G. Visualizing data using t-SNE. *J. Mach. Learn. Res.* **9**, 2579-2605 (2008).
